# Prevalence of Infection and Antibiotic Susceptibility of *Helicobacter pylori*: An Evaluation in Public and Private Health Systems of Southern Chile

**DOI:** 10.3390/pathogens8040226

**Published:** 2019-11-09

**Authors:** Marcelo Oporto, Monica Pavez, Claudia Troncoso, Alvaro Cerda, Edmundo Hofmann, Armando Sierralta, Eddy Rios, Luis Coppelli, Leticia Barrientos

**Affiliations:** 1 Center of Excellence in Translational Medicine, CEMT-BIOREN, Universidad de La Frontera, Temuco 4810296, Chile; m.oporto01@ufromail.cl (M.O.); alvaro.cerda@ufrontera.cl (A.C.); leticia.barrientos@ufrontera.cl (L.B.); 2 Doctoral Program in Sciences, Specialty in Applied Cellular and Molecular Biology, Universidad de La Frontera, Temuco 4811230, Chile; troncosomunozc@gmail.com; 3 Faculty of Science Health, Universidad Autónoma de Chile, Temuco 4800913, Chile; 4 Department of Internal Medicine, Universidad de La Frontera, Temuco 4781218, Chile; edmundo.hofmann@ufrontera.cl (E.H.); armando.sierralta@gmail.com (A.S.); eddy.rios@ufrontera.cl (E.R.); 5 Gastroenterology Unit of Clinica Alemana de Temuco, Temuco 4810297, Chile; 6 Gastroenterology Unit of Hospital Hernan Henriquez Aravena, Temuco 4781151, Chile; 7 Gastroenterology Unit of Hospital de Villarrica, Villarrica 4930000, Chile; luis.coppelli@gmail.com

**Keywords:** prevalence of *Helicobacter pylori* infection, public and private health systems, antibiotic resistance

## Abstract

*Helicobacter pylori* colonizes half of the human population. Age, ethnicity, and socioeconomic status are factors that influence the prevalence of the infection. This is important in southern Chile, one of the most unequal regions in the world, where a significant difference in the health access of the population occurs due to the existence of two competing health systems. Moreover, in the last few years, current protocols of *H. pylori* eradication have shown high rates of resistance with reduced therapeutic efficacy. This study reported the epidemiology of infection and attempted to identify divergent points among the population beneficiaries of the two health care schemes in southern Chile. Biopsies from public (n = 143) and private (n = 86) health systems were studied. At the same time, clinical and sociodemographic factors were evaluated. *H. pylori* strains were obtained from gastric biopsies for culture and molecular testing. Antibiotic susceptibility was determined by the agar dilution method. Differences about ethnicity, rural residence, and education (*p* ≤ 0.05) were observed between beneficiaries of the two health systems. The prevalence of *H. pylori* was 45%, with no significant differences regardless of the socioeconomic conditions. The only identified risk factor associated with *H. pylori* infection was Mapuche ethnicity (OR (odds ratio) = 2.30). *H. pylori* showed high resistance rates, particularly against clarithromycin (40%), levofloxacin (43.1%), and metronidazole (81.8%). This study highlighted the importance of Mapuche ancestry as a risk factor in southern Chile and emphasized the need to search for new eradication strategies as well as further studies evaluating therapeutic efficacy.

## 1. Introduction

*Helicobacter pylori* (*H. pylori*) infection is a serious public health problem, colonizing more than half of the human population, with a prevalence of 40–50% in developed countries and up to 90% in developing countries. In Chile, the prevalence of infection varies between 40% and 70%, according to the World Organization of Gastroenterology (WGO) and reports of some national studies [1,2]. Age, ethnicity, gender, geography, and socioeconomic level are factors that influence the incidence and prevalence of the infection [2]. A study conducted in six Latin American countries observed that prevalence of *H. pylori* infection varied between 70% and 84% depending on the country and particular living conditions, such as overcrowding, presence of dirt floors, etc. The study closely linked *H. pylori* infection with a low socioeconomic level of the population, increasing the risk of infection [3]. This also acquired significance considering the type of health care system available. In Latin America, one of the most unequal regions in the world, health systems coexist and are in direct competition with each other, conditioned, on the payer/provider split, free choice, and pre-paid health service plans [4]. In Chile, the delivery of health services relies on public or private health attendance. The distribution of Chilean population by type of health provider shows a clear socioeconomic-split, with high-income low-risk people covered predominantly by private health services, while low-income high-risk people rely on public sector coverage [5].

On the other hand, the eradication treatment based on a triple therapy scheme (proton-pump inhibitor (PPI) plus two antibiotics) recommended by the Maastricht consensus is the currently most used worldwide [6]. However, in the last few years, worryingly high rates of *H pylori* resistance to several antibiotics used in eradication treatments have been reported. Moreover, the variation among geographic areas in the same country makes it difficult to generalize effective therapies in some populations [7,8]. Antibiotic resistance is a key factor in the failure of the eradication treatment and the persistence of *H. pylori* infection. Several susceptibility studies have been carried out to evaluate treatment efficacy, mainly for clarithromycin, where the therapeutic success rate could decrease to 40% or less in the event of resistance to this antibiotic [9,10]. Implementation of new eradication strategies requires up-to-date information regarding the prevalence of *H. pylori,* its antibiotic susceptibility, and all associated factors previously described [11].

Gastric cancer is one of the most frequent neoplasia in the Chilean population, and it is the leading cause of death from malignant tumors in both sexes, with an overall mortality rate of 20 per 100,000 inhabitants. This pathology and its association with *H. pylori* infection are a public health priority for the country [12]. Southern Chile, despite its high prevalence of stomach diseases and high gastric cancer mortality rate, lacks data and studies in *H. pylori* prevalence and antibiotic susceptibilities. The present study aimed to update the current epidemiology of the infection and to identify divergent points of our population in relation to the two health care systems, providing guidelines to prioritize and customize public health efforts for better management of *H. pylori* infection in our population.

## 2. Results

During the study period, 229 gastric tissue samples (antrum biopsies) were obtained from dyspeptic patients during endoscopy procedures carried out at public (Regional Hospital of Temuco, HHHA and Villarrica Hospital, HV) and private (Clinica Alemana of Temuco, CAT) health systems, of which 143 (62.44%) were from the public service and 86 (37.55%) from the private service (Table 1). The average age of the study population was 50.68 years, with a predominance of females. The attention of the Mapuche population (a local ethnic group of the Araucanía) was significantly greater in the public health system (*p* < 0.05). Similarly, rural residence, education level <12 years, and national public health insurance (known as FONASA—“FOndo NAcional de SAlud”) were also higher in this group, demonstrating points of divergence between the two populations, with more vulnerable sociodemographic characteristics in the public system. On the other hand, private service had a greater number of patients who drank alcohol (*p* < 0.05). It was also noted in the clinical histories that the private health system patients had a greater presence of a dyspeptic population previously diagnosed with *H. pylori* infection.

Of all patients, 45.41% were positive for the urease test, 44.18% in the private service, and 46.15% in the public service, with no statistically significant differences between them. The *Helicobacter pylori* was identified in 90 patients with a positive urease test, observing a recoverability of 87% from biopsy samples (Table 2).

Since no differences were observed in the prevalence of *H. pylori* infection between private and public systems, the analysis of the influence of sociodemographic characteristics on *H. pylori* infection was assessed in total population, regardless of the health system attendance. An analysis of the sociodemographic characteristics between the infected and non-infected patients (Table 3) revealed that the variable associated with the Mapuche ethnic group presented a statistically significant difference and was associated with an increased risk of infection (OR (odds ratio) = 2.30, CI = 1.14–4.78, *p* = 0.026).

Forty-four *H. pylori*-positive samples with molecular confirmation were selected for the antibiotic susceptibility study. The overall resistance study was performed in 41 strains of treatment-naive patients, and three strains of patients with previous treatments for *H. pylori* eradication. High percentages of strains resistant to amoxicillin (minimum inhibitory concentration, MIC_50_ = 0.125 µg/mL, MIC_90_ >32 µg/mL), clarithromycin (MIC_50_ = 0.125 µg/mL, MIC_90_ > 32 µg/mL), levofloxacin (MIC_50_ = 1 µg/mL, MIC_90_ = 16 µg/mL), and metronidazole (MIC_50_ and MIC_90_ > 32 µg/mL) were observed (Table 4). The only resistance rate slightly lower was against tetracycline (MIC_50_ = 0.5 µg/mL, MIC_90_ = 2 µg /mL) (Table 4). On comparing the antibiotic resistance in both health systems, the only significant difference was observed against levofloxacin, which was higher in the public system (56.6% vs. 14.3%, *p* = 0.031).

The primary resistance profile of treatment-naive patients was very similar to those observed in the overall resistance rates, with a slight difference only in clarithromycin (n = 16.39%). Secondary resistance to previous treatment for clarithromycin and levofloxacin was observed in two and one isolates, respectively. Interestingly, one isolate of a patient with previous treatment was susceptible to all drugs, except metronidazole.

## 3. Discussion

The present study contributed to the limited number of studies on prevalence and risk factors associated with *H. pylori* infection in the Región de La Araucanía and Chile, in addition to providing a preliminary view of the antibiotic susceptibility profile of this bacterium. Región de La Araucanía, one of the regions with the highest gastric cancer mortality rates in Chile [13], is characterized by having a socioeconomic vulnerability that may be related to the risk factors extensively associated with *H. pylori* infection. As such, knowledge of the prevalence and antibiotic susceptibility profile of the bacterium in this region is vitally important for determining strategies to manage and treat the infection.

The sociodemographic characteristics of the sample population (Table 1) showed that the average age of the study population was 50.68 ± 16.85 years, with females predominating (64.17%). The public health service had higher values of sociodemographic variables, such as Mapuche ethnicity (*p* = 0.025), rural residence (*p* = 0.05), education level ≤12 years (*p* < 0.001), and low management of *H. pylori* infections, as compared to private system. These differences allowed us to categorize the group attended in the public health system in the region as being on a lower socioeconomic level, factors that predispose individuals to an increased risk of *H. pylori* infection [14]. On the other hand, patients from the private health system had a higher prevalence of alcohol consumption, considered to be a risk factor for the infection and the progression of lesions. It is important to highlight that previous evidence linked alcohol consumption to *H. pylori* infection mainly by being related to poor lifestyle habits of the host [2,15]. In this line, these results might be biased as differences between the amounts of alcohol ingested by each patient were not established in this study.

The differences in sociodemographic characteristics between health systems observed in this study were in agreement with the distribution of the population in the different health care systems in Chile. Chile has a health system, in which the benefits depend mostly on their income, so the accessibility to private health care systems is based on co-payments, as defined according to their health insurance policies [16]. Thus, the population served in the private health system, which belongs mostly to the two highest quintiles of the population, has access to a system that provides faster attention and better infrastructure, keeping easy access to public emergency and intensive care treatment. The population with the lowest income only has the public option, with a more deficient and slow primary health care, and poor medical infrastructure [5,16].

### 3.1. Prevalence of H. pylori Infection

The prevalence of *H. pylori* infection is greater in developing countries, reaching 95% and 30% to 50% in developed countries [2,17]. Globally, Africa, South America, and Western Asia have the highest prevalence rates of infection in their populations, with 70.1%, 69.4%, and 66.6%, respectively [2]. A meta-analysis that simultaneously compared the prevalence of the infection in seven countries determined a prevalence of 76.8% in Chile in 2013 using the urea breath test. Ferreccio et al., in 2007, reported a similar value, with a prevalence of 72.99%. Both studies were conducted on adult populations in the Metropolitan Region of Chile [3,18].

In the year 2000, in the Región de La Araucanía, a prevalence of 82% was reported in a retrospective study of 200 patients in the HHHA; however, that study focused on patients with a histopathological report of chronic gastritis and not on dyspeptic patients [19]. Interestingly, the present study showed a significantly lower prevalence of active infection in the region (45.41%), differing considerably with previous reports on the population. This decrease may be related to several factors, such as to the improvement in the sanitary conditions and antibiotic use by the population [17]. Da Costa et al. observed a 16% reduction in prevalence on patients at the University of Chile’s Clinical Hospital in a period of 11 years, which was mainly attributed to factors like the universal indication of eradication treatment to all patients infected by *H. pylori* and improvements in the socio-economic and hygiene levels of population [20]. In fact, previous *H. pylori* infection reported from clinical histories of patients showed the presence of this bacterium in at least 58.5% (n = 134) of the total population, with a 23.13% (n = 31) of these patients previously infected and treated, and only 20 of them with successful eradication (64.5%). Interestingly, there was a difference in the management of *H. pylori* infection between health service systems. Patients from the public system showed a low treatment rate in previously infected patients, a fact possibly associated with poor access to the eradication therapy within this health system.

An important finding in our study group was the colonization by the bacterium, which was independent of the population’s socioeconomic differences observed between public and private health systems, despite these factors have been widely reported as risk factors for the infection [21,22,23,24,25]. These results implied that these divergent points were not associated with an increased risk of *H. pylori* colonization in the Región de La Araucanía.

In the multivariate analysis evaluating contributing factors for *H. pylori* infection, it was determined that belonging to the Mapuche ethnic group was the only variable that represented a risk factor associated with *H. pylori* infection. Mapuche is an indigenous population of greatest prevalence in Chile, mainly in the Región de La Araucanía, who had approximately 2.3 times increased risk of infection in this study (OR: 2.30; CI: 1.14–4.78; *p* = 0.026).

There are scarce studies about genetic components associated with colonization or the acquisition of *H. pylori* infections. Previous studies evaluating major histocompatibility complex (MHC, Human Leukocyte Antigens -HLA-) class II genes have suggested a role of the surface receptor HLA-DRB1 alleles in susceptibility and maintenance of *H. pylori* infection in Japanese and European populations [26]. In the US, the differences in the prevalence between the white and black populations have also been widely described; in 1991, Graham et al. obtained a prevalence in the black population almost to double to that of the white population [27]. Similarly, in 2002, Malaty et al. determined by multivariate analysis that being black was a risk factor for the infection, although the socioeconomic factor of the population was not assessed [28]. In South America, Amerindian indigenous communities are characterized by lower socioeconomic levels, associated with poor living conditions, favoring transmission routes in these minority ethnic groups. However, in this study, the association of ethnicity with *H. pylori* infection was significantly greater in patients who were attended in the private health system (*p* = 0.029) (data not shown), where there was no association with vulnerable risk factors for the infection. Chile has a high percentage of people of Amerindian ancestry, particularly in the Región de La Araucanía due to a large number of Mapuche descendants [29]. Therefore, the results observed could be of great significance in the country, although further genetic studies are required.

This small-scale descriptive epidemiology study was based on the inclusion of patients consulting by dyspeptic symptoms between January to December 2018 from major health care centers of the Región de La Araucanía. Despite the small sample size, the main result reported was the relationship between *H. pylori* infection and Mapuche ancestry that showed a prevalence of 30.76% and 19.2% of Mapuche ancestry in *H. pylori*-infected and non-infected groups, respectively. With a 5% value of alpha, and considering the number of individuals included (n = 229), the statistical power was 82%. Although statistical power supported the differences found according to Mapuche ancestry in this study, our conclusions should be carefully interpreted and not generalized for the entire population in Chile.

### 3.2. Antibiotic Susceptibility

Antibiotic resistance is a key factor in the failure of the eradication treatment and the recrudescence of *H. pylori* infection. Several susceptibility studies have been carried out to evaluate the efficacy of treatments in different regions of the world, mainly for clarithromycin, where the therapeutic success rate decreases to 40% and up to 85% if there is resistance to this antibiotic [9,10]. In Chile, the eradication rates of *H. pylori* reached up to 84% in 2011 [30]; however, a recent study performed by our research group observed an eradication rate between 71% and 79% in patients from La Araucanía [31], numbers below of the current national guidelines (90%) [2]. In fact, according to data of clinical histories of the patient, the eradication rate observed in our research was 65.9%, slightly lower to observed by Hofmann et al. [31].

Currently, the most used treatment in the eradication of *H. pylori* is the triple therapy, which includes PPI, clarithromycin plus amoxicillin or nitroimidazoles. According to the guidelines of the V Maastricht consensus, the culture and the study of phenotypic and genotypic susceptibility have begun to be used as a selection guide, mainly for areas with high resistance rates. According to these same recommendations, the use of clarithromycin without previous susceptibility studies must be reviewed in regions where resistance to this antibiotic is >15%. Similarly, the use of metronidazole must be reviewed in a region with high rates of resistance, and first-line quadruple therapies with bismuth are recommended for its replacement [6].

The overall resistance rate observed for clarithromycin was 40.9%, greater than those reported by other authors in the country, which reported overall resistance rates of 2.2% and 20% in two studies conducted in central areas of Chile [32,33]. However, both studies concluded that resistance to clarithromycin was increasing in the country. Already in Europe, Megraud et al. (2013) reported a primary resistance rate in the adult population of 17.5% in 2013 [34]. Moreover, overall resistance rates to clarithromycin have reached 30% in Italy and Japan, 40% in Turkey, and 50% in China, although rates in Sweden and Taiwan have been reported only 15% [35]. Even though susceptibility to clarithromycin is regionally variable, every area in the world that has been studied on more than one occasion shows increasing resistance rates to antibiotics, both in high and middle/low-income countries, reducing the efficiency of the treatment [6]. One of the possible causes of this increase in macrolide resistance could be the broad use of these antibiotics in the treatment of various infections of the respiratory tract and others [36]. Megraud et al. observed a significant association between the use of long-acting macrolides and clarithromycin resistance (*p* = 0.036) [34].

In this study, we also observed a strong resistance to metronidazole (81.8%). Globally, there are other regions with high primary resistance rates, such as 39.9% in developed countries from Western Europe, 81% in India, 42% in the US, and 96.4% in Southeast Asia [34,37,38,39]. In Chile, in 2007, a primary resistance of 44.9% for Metronidazole was reported in the Región del Bio-Bio and overall resistance of 41.8% in the Region Metropolitana in studies conducted on adult populations [32,33]. It is important to point out that antibiotic resistance rates to clarithromycin and metronidazole, according to Maastricht consensus guidelines, would not allow us to use them as first-line treatments in empirical therapy [6], making necessary a reconsideration of national guidelines for *H. pylori* eradication.

Regarding levofloxacin susceptibility, the overall resistance rate was 43.1%, a slightly higher value than those reported in Latin America, with primary resistances of 23% in Brazil and overall resistance rates of 36.9% in Peru [40,41]. Worldwide resistance rates of 14.1% for Europe and 11.6% in Asia have been reported, observing a direct association between consumption of quinolones and resistance rate of Levofloxacin [7,34]. The broad use of quinolones in the treatment of several infections favors the exposition of *H. pylori* to selective pressure and allows it to evolve quickly, incrementing the rate of resistance to the antibiotic [34]. The analysis of the resistance profile obtained from *H. pylori* determined that only levofloxacin showed statistically significant differences between health systems (*p* = 0.007), having higher rates in strains isolated from patients of the public than those of the private sector. This was the first report of susceptibility to levofloxacin for *H. pylori* in the country.

Finally, no considerably high resistance to tetracycline and amoxicillin has been reported worldwide. Recent studies in Europe have shown primary resistance to tetracycline of 2.5% in Germany and 0% in northern Spain [42,43]. In China, a primary resistance of 15% was reported in the city of Zhuanghe in 2019 [44]. In our study, a similar resistance rate (13.6%) was observed, lower than the 26.8%, previously reported in Chile [33]. When amoxicillin susceptibility was evaluated, we found a higher than expected rate of overall resistance (11.3%). The resistance rate in Latin America is similar to that of European regions [45]. An overall resistance of 1.9% has been reported in patients with dyspeptic syndrome from Brazil [46]. This last value is in line with the report by González et al., which described 0% of resistant strains in Concepción-Chile [32]. Interestingly, the duality of resistance rates was observed in this article for amoxicillin due to discordant cutoff proposed by the literature for Latin America [45] and the European Committee on Antimicrobial Susceptibility Testing (EUCAST) [47]. This difference demonstrated the lack of clear guidelines on monitoring studies for the treatment of *H. pylori*, showing that there is insufficient clinical and scientific evidence to delineate the cutoff points for amoxicillin, metronidazole, levofloxacin, and tetracycline in the eradication of *H. pylori* [48,49]. Larger studies are necessary to find the correlation of efficacy with the different levels of antibiotic susceptibility, mainly in Latin-America.

## 4. Materials and Methods

### 4.1. Patient Recruitment

This cross-sectional study was carried out between January and December 2018. A total of 229 dyspeptic patients who underwent endoscopy at public (Regional Hospital of Temuco, HHHA and Villarrica Hospital, HV) and private (Clinica Alemana of Temuco, CAT) health systems, were included in the study after signing the informed consent. The study design and informed consent were previously approved by the Bioethical Committee of Universidad de La Frontera, Chile (Number protocol: 109/2016). All patients were over 18 years old, and they had not taken antibiotics and proton pump inhibitors within three weeks prior to endoscopy. Before sample collection, individuals were questioned about their sociodemographic characteristics, such as their highest level of education, main occupation, gender, age, race, Mapuche ethnicity, and origin. Questions about clinical history included current medications, history of gastric cancer, history of *H. pylori* infection, and treatments for the bacteria to evaluate previous management of *H. pylori*.

### 4.2. Sample Collection

A biopsy was taken from the patient’s stomach during the endoscopy procedure. The urease test results were recorded in the respective health service (HHHA, HV, and CAT). The biopsy taken from the antrum was stored in cryopreservation tubes with 200 µl of Brucella broth (BD-DIFCO, Berkshire, UK) and transported at 4 °C to the Applied Molecular Biology Laboratory of the Universidad de La Frontera for culture and identification. In addition, the sample taken for the urease test was also collected for the molecular confirmation of the presence of *H. pylori.*

### 4.3. Culture and Phenotypic Identification of H. pylori

The biopsy sample was macerated, homogenized, and inoculated in Columbia blood agar (BD-DIFCO, Berkshire, UK), supplemented with 7% horse blood and selective antibiotics for *H. pylori*, such as trimethoprim, amphotericin B, and polymyxin B in concentrations of 5 µg/ml each and vancomycin 10 µg/ml (DENT supplement, OXOID Limited, Hampshire, UK). The incubation period was 5–10 days at 37 °C in microaerophilic conditions (5% O_2_, 10% CO_2_, 85% N_2_, and 90% humidity) using commercial systems (CampyGen, OXOID Limited, Hampshire, UK). The suggestive growth of *H. pylori* was identified through phenotypic testing of oxidase and catalase and morphological characteristics of the bacterium by Gram staining. The strains suggestive of *H. pylori* were suspended in Columbia broth with 5% horse serum and 20% glycerol to be stored at −80 °C.

### 4.4. DNA Extraction from Pure Culture

DNA was extracted from isolated strains using the commercial kit Ultra Clean® Microbial DNA Isolation Kit (MO BIO Laboratories, Carlsbad, CA, USA) according to the manufacturer’s instructions. The extracted DNA was stored at −20 °C.

### 4.5. Total DNA Extraction from Urease Biopsy

Biopsy samples from the urease test were used for total DNA extraction, using the method modified by Baroni et al. [50]. Briefly, 400 μL of TES buffer with 2 μL of proteinase k (20 mg/mL) was added to the tissue, and the tubes were incubated at 60 °C for 16 h. Then, 125 μL of NaCl (5 M) and 400 µL of chloroform were added. The tubes were shaken until a milky solution was formed. The tubes were centrifuged at 10,000× *g* for 3 min, and the upper aqueous phase was transferred to a new tube. An equal volume of cold ethanol was added at 95%, and the tubes were inverted 3 times to condense the DNA. Centrifugation at 10,00× *g* was carried out for 2 min, and the supernatant was eliminated. The sediment was washed with 1 ml of cold 70% ethanol, and each tube was centrifuged for 1 min at 10,000× *g*. The supernatant was discarded, and the pellet was re-suspended in 50–200 µL of TE buffer (pH 8) to be conserved at −20 °C.

### 4.6. Species Confirmation by Molecular Biology

The extracted DNA was used for the molecular confirmation of *H. pylori*. This was done by PCR amplification of the UreC gene, using the following primers: FExt: 5′- AGCTATAAAGTGGGCGAGAG-3, RExt: 5′-ATTGCCCGTTAGGCTCAT-3. All the amplifications were done under conventional PCR conditions described through the method modified by Wormwood (2000) [51]. The PCR products were visualized by 1% agarose gel electrophoresis.

### 4.7. Antibiotic Susceptibility Study

Antibiotic susceptibility was evaluated by the agar dilution method to determine the minimum inhibitory concentration (MIC) of the antibiotics amoxicillin, clarithromycin, metronidazole, tetracycline, and levofloxacin according to the Clinical and Laboratory Standards Institute (CLSI) M100 guidelines [52]. MIC values were determined based on the 9th version of the EUCAST clinical breakpoint (2019) [47], considering as strains resistant to amoxicillin concentrations >0.125 µg/mL, >0.5 µg/mL for clarithromycin, >1 µg/mL for levofloxacin, >8 µg/mL for metronidazole, and >1 µg/L for tetracycline. For amoxicillin, the MIC was also determined according to values from bibliographical references considering strains resistant to concentrations >2 µg/mL. *H. pylori* ATCC 43504 was used as a quality control strain.

### 4.8. Statistical Analysis

The statistical analyses were performed in the program Minitab®18. The chi-square test of association was used to establish differences between the sociodemographic characteristics and to compare the presence of *H. pylori* between the study groups (public and private health services). The established degree of significance was *p* < 0.05. To determine variables associated with the risk of developing the *H. pylori* infection, binary logistic regression models were adjusted, obtaining the odds ratio (OR) and its 95% confidence intervals.

## 5. Conclusions

Despite the sociodemographic differences of the populations studied between public and private systems in the Región de La Araucanía, the prevalence of *H. pylori* infection was similar among the affected populations, with an average of 45%. The only risk factor for infection observed in the total population, independent of socioeconomic conditions, was the Mapuche ancestry, a result of great concern for Southern Chile, although further genetic studies are required.

The resistance profiles observed in this study confirmed the increasing resistance rates of *H. pylori* against several antibiotics. This suggested the need to seek new eradication strategies as well as further studies evaluating therapeutic efficacy in this geographic area before establishing an empiric use of an eradication program.

## Figures and Tables

**Table 1:** Sociodemographic characteristics of all the patients according to the health service system.

Variables	Total, n = 229 (n (%))	Private, n = 86 (n (%))	Public, n = 143 (n (%))	*p*-Value
Age (years)	50.68 ± 16.85	46.27 ± 15.82	53.20 ± 17.02	0.344
Sex (male/female)	82 (35.80%)/147 (64.17%)	34 (39.53%)/52 (60.46%)	48 (33.56%)/95 (66.43%)	0.362
Mapuche ethnicity	53 (23.14%)	13 (5.11%)	30 (20.97%)	0.025*
Rural residence	44 (19.21%)	11 (12.79%)	33 (23.07%)	0.050*
Education level ≤12 years	132 (57.64%)	26 (30.23%)	106(74.12%)	<0.001*
National health insurance (FONASA)	188 (82.09%)	48 (55.81%)	140 (97.90%)	<0.001*
Family group ≥5 members	29 (12.66%)	11 (12.79%)	18 (12.58%)	0.964
Smoker	57 (24.89%)	27 (31.39%)	30 (20.97%)	0.129
Drinker	104 (45.41%)	58 (67.44%)	46 (32.16%)	<0.001*
Previous infection	52 (22.70%)	25 (29.06%)	27 (18.88%)	0.075
Previous treatment	31 (13.53%)	17 (19.32%)	14 (9.93%)	0.046*
Relative with GC	76 (33.18%)	30 (34.88%)	46 (32.16%)	0.673

GC: Gastric cancer. Age expressed as average ± SD (Range). Categorical variables expressed as counts and percentages. * Statistical significance (*p* < 0.05). FONASA, FOndo NAcional de SAlud.

**Table 2:** Results of urease tests and prevalence of *H. pylori* obtained by culture.

	Total, n = 229 (n (%))	Private, n = 86 (n (%))	Public, n = 143 (n (%))	*p*-Value
Urease positive	104 (45.41%)	38 (44.18%)	66 (46.15%)	0.592
*H. pylori* positive	90 (39.30%)	32 (37.20%)	58 (40.55%)	0.472

**Table 3:** Sociodemographic characteristics of all the patients, according to *H. pylori* infection.

Background	Infected Patients n = 104 (n (%))	Non Infected Patients n = 125 (n (%))	Odds Ratio (CI 95%)	*p*-Value
Age (years)	51.16 ± 16.85	52.97 ± 15.91	0.98 (0.96–1.00)	0.095
Sex (male/female)	34 (32.69%)/70 (67.30%)	48 (38.04%)/77 (61.60%)	0.71 (0.38–1.34)	0.303
Mapuche ethnicity	32 (30.76%)	24 (19.20%)	2.30 (1.14–4.78)	0.026*
Rural residence	21 (20.19%)	21 (16.80%)	1.00 (0.43–2.33)	0.982
Education level ≤12 years	59 (56.73%)	68 (54.40%)	0.70 (0.34–1.47)	0.354
National health insurance (FONASA)	82 (78.84%)	94 (75.20%)	1.33 (0.55–3.17)	0.519
Family group ≥5 members	11 (10.57%)	17 (13.60%)	0.52 (0.21–1.28)	0.161
Smoker	26 (25.00%)	28 (22.40%)	1.18 (0.58–2.37)	0.642
Drinker	50 (48.07%)	48 (38.40%)	1.26 (0.66–2.38)	0.470
Previous infection	19 (18.26%)	30 (24.00%)	0.62 (0.23–1.69)	0.360
Previous treatment	11 (10.57%)	20 (16.00%)	0.91 (0.27–3.08)	0.887
Relative with GC	38 (36.53%)	37 (29.60%)	1.07 (0.57–2.01)	0.811

GC: Gastric cancer. Age expressed as average ± SD (Range). Categorical variables expressed as counts and percentages. * Statistical significance (*p* < 0.005).

**Table 4:** Antibiotic resistance profile for isolates of *H. pylori.*

Antibiotics	Total, n = 44 (n (%))	Private, n = 14 (n (%))	Public, n = 30 (n (%))	*p*-Value
**Amoxicillin**	16 (36.30%)/5 (11.30%) *	3 (21.30%)*	2 (6.90%)*	0.164
**Clarithromycin**	18 (40.90%)	8 (57.10%)	10 (33.30%)	0.149
**Levofloxacin**	19 (43.10%)	2 (14.30%)	17 (56.60%)	0.031**
**Metronidazole**	36 (81.80%)	12 (85.70%)	24 (80.00%)	0.607
**Tetracycline**	6 (13.60%)	2 (14.30%)	4 (13.30%)	0.932

Categorical variables expressed as percentages. * Cutoff point >2 µg/ml. ** Statistical significance (*p* < 0.05).
